# Tpr regulates the total number of nuclear pore complexes per cell nucleus

**DOI:** 10.1101/gad.315523.118

**Published:** 2018-10-01

**Authors:** Asako McCloskey, Arkaitz Ibarra, Martin W. Hetzer

**Affiliations:** Molecular and Cell Biology Laboratory, The Salk Institute for Biological Studies, La Jolla, California 92130, USA

**Keywords:** nucleus, nuclear envelope, nuclear pore complex, nucleoporin, Tpr, Nup153, ERK

## Abstract

The total number of nuclear pore complexes (NPCs) per nucleus varies greatly between different cell types and is known to change during cell differentiation and cell transformation. However, the underlying mechanisms that control how many nuclear transport channels are assembled into a given nuclear envelope remain unclear. Here, we report that depletion of the NPC basket protein Tpr, but not Nup153, dramatically increases the total NPC number in various cell types. This negative regulation of Tpr occurs via a phosphorylation cascade of extracellular signal-regulated kinase (ERK), the central kinase of the mitogen-activated protein kinase (MAPK) pathway. Tpr serves as a scaffold for ERK to phosphorylate the nucleoporin (Nup) Nup153, which is critical for early stages of NPC biogenesis. Our results reveal a critical role of the Nup Tpr in coordinating signal transduction pathways during cell proliferation and the dynamic organization of the nucleus.

## Abstract (precis)

In this study, McCloskey et al. investigated the underlying mechanisms that control how many nuclear transport channels are assembled into a given nuclear envelope. Their results show that depletion of the NPC basket protein Tpr, but not Nup153, dramatically increases the total NPC number in various cell types and provide insight into a critical role of the nucleoporin Tpr in coordinating signal transduction pathways during cell proliferation and the dynamic organization of the nucleus.

Nuclear pore complexes (NPCs) are essential nuclear transport channels that span the nuclear envelope (NE). In addition to their canonical role in nucleo–cytoplasmic traffic, NPCs interact directly with the genome to regulate chromatin organization and developmental gene expression (Ibarra and Hetzer 2015; Ibarra et al. 2016; Raices et al. 2017). How many NPCs are assembled per nucleus is tightly controlled in a cell type-specific manner (Maul and Deaven 1977). In mammalian cells, the total NPC number can vary from a few hundred to tens of thousands of NPCs (Maul and Deaven 1977; Garcia-Segura et al. 1989). Importantly, the differences in NPC number are not directly linked to available NE surface area, as the density also varies among cell types (Maul and Deaven 1977). Furthermore, NPC numbers change through differentiation, such as for neural progenitor cells, which give rise to multiple neuronal cell types with significantly different NPC numbers (Garcia-Segura et al. 1989; Toda et al. 2017). Recent evidence suggests that changes in NPC numbers are critical for cell differentiation (Jacinto et al. 2015). A failure to properly regulate NPC numbers has also been linked to aggressive tumorigenesis and activation of lymphocytes or thyroid cells (Many et al. 1981; Czerniak et al. 1984). All of these studies suggest that the rate of NPC assembly is under cellular control. However, despite the importance of NPC homeostasis for cell function, the molecular mechanism by which cells determine the NPC number remains a mystery.

NPCs are highly stable structures once they are assembled into the NE of dividing cells (Rabut et al. 2004; Toyama and Hetzer 2013); thus, their overall numbers are likely to be controlled at the assembly stage. Formation of new NPCs occurs at different stages of the cell cycle: (1) directly at the end of mitosis during the reformation of the NE and (2) in interphase into an intact NE. During mitosis, cells disassemble the NE and NPCs in prophase, followed by a rapid but coordinated reassembly, which starts at late anaphase (Burke and Ellenberg 2002). This step requires the nucleoporin (Nup) Elys, one of the scaffold components of NPCs. Elys binds to the chromatin/nuclear periphery in late anaphase and initiates the post-mitotic NPC assembly by recruiting the crucial core components of NPCs (the Nup107/160 complex) (Franz et al. 2007). In interphase, cells double the NPC number from G1 to G2 phase by a slow assembly, which inserts NPCs into the intact NE (Doucet et al. 2010; Dultz and Ellenberg 2010). One of the membrane Nups, Pom121, was shown to establish a membrane platform for future NPC assembly before Nup107/160 complex recruitment, while Elys is not required (Doucet et al. 2010; Funakoshi et al. 2011). Interestingly, it has been shown recently that one of the nuclear basket components, Nup153, plays an important role in interphase NPC assembly by binding directly to the inner nuclear membrane (INM) and recruiting the Nup107/160 complex to NPC assembly sites (Vollmer et al. 2015). Furthermore, a recent study based on electron microscopy has shown a large invagination at the INM, indicating a possible NPC intermediate assembling in interphase (Otsuka et al. 2016). These studies implied that Nups at the nuclear side might be the key components of early interphase NPC assembly, providing an interesting possibility that cells might control NPC assembly through these Nups.

Here, we show that Tpr, one of the nuclear basket Nups, but not Nup153, is a crucial factor to negatively regulate NPC assembly. We demonstrated that phosphorylation of Tpr and Nup153 by extracellular signal-regulated kinase (ERK), which is an essential serine/threonine kinase of the mitogen-activated protein kinase (MAPK) pathway, plays an important role in recruiting the Nup107/160 complex to the NE.

## Result

### Depletion of Tpr increases NPC numbers

**Figure 1.**
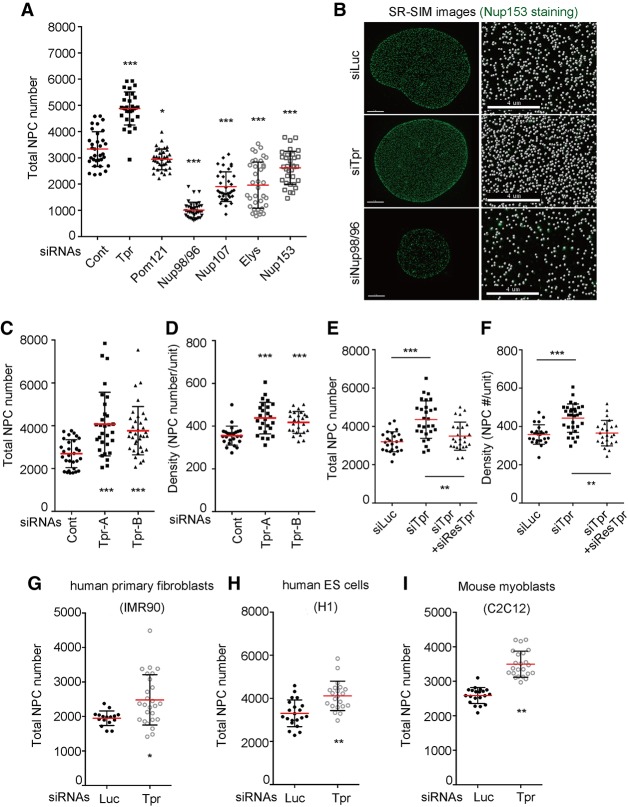
Knockdown of Tpr increases the NPC numbers. To count NPC numbers, all of the images were taken by SR-SIM and analyzed by Imaris software. Mean values of NPC numbers are indicated by red bars. *t*-test was performed to obtain *P*-value. (*) *P* < 0.01; (**) *P* < 0.001; (***) *P* < 0.0001. (*A*) After knockdown of various Nups for 72 h, nuclei were stained with Nup153, Elys, or mAb414. SR-SIM images of nuclei were taken, and the NPC numbers were obtained by counting Nup153 (for knockdown of Luc, Tpr, Pom121, Nup98/96, Nup107, and Elys) and Elys (for knockdown of Nup153). (*B*, *left* column) U2OS nuclei were stained with Nup153, and images were obtained using SR-SIM. Bars, 4 µm. (*Right* column) Enlarged SR-SIM images. The center regions of nuclei in the *left* column are enlarged to show the density of NPCs. White spheres represent Nup153 foci. Bars, 4 µm. (*C*) U2OS cells were transfected with two different siRNAs against endogenous Tpr. The total NPC numbers were obtained by counting Nup153 foci. More than 30 nuclei were analyzed. (*D*) The density increase in Tpr knockdown conditions using either of siRNAs. The density was obtained by counting NPC numbers using SR-SIM in the rectangular volume created by 150 pixels × 150 pixels × nuclear height, which allowed us to count NPCs in 150-pixel × 150-pixel areas at both sides of the nucleus. The rectangular volumes were carefully located in the center of the nuclei to avoid the overestimation of NPC numbers. More than 30 nuclei were analyzed. (*E*,*F*) The rescue experiment expressing an siRNA-resistant Tpr recombinant construct. The plasmid encoding siRes-Tpr was delivered simultaneously with siRNA against endogenous Tpr. Total NPC number (*E*) and the density (*F*) were obtained by counting Nup153 foci using SR-SIM. (*G*–*I*) Tpr was depleted from IMR-90, C2C12, and human embryonic stem cells. Total NPC numbers were counted using Nup153 foci using SR-SIM.

Once they are assembled into the NE, NPCs are remarkably stable structures that are not removed during all of interphase (Rabut et al. 2004). We therefore reasoned that any mechanism that controls total NPC numbers is likely to occur at early NPC assembly steps. The formation of a new NPC is a multistep process that involves the chromatin-binding Nup Elys, the transmembrane protein Pom121, and the multimeric Nup107/160 complex. As shown recently, the nuclear basket component Nup153 targets the assembly process to the NE (Vollmer et al. 2015). Consistent with their role in NPC assembly, we found that the depletion of any of these Nups blocked the formation of new NPCs and resulted in a dramatic decrease in NPC number as determined by superresolution structured illumination microscopy (SR-SIM) (Fig. 1A,B, top and bottom panels). These results confirm previous studies and support the notion that multiple Nups are required to coordinate the recruitment of ∼30 different polypeptides to a new NE assembly site.

Tpr, another nuclear basket component, is a Nup that stands out in terms of its recruitment to the NPCs. While almost all other Nups are incorporated into the NE at the end of mitosis, Tpr is the last Nup to be added to NPCs in early G1 (Bodoor et al. 1999; Rajanala et al. 2014). We therefore wondered whether depletion of Tpr had an effect on NPC assembly. To our great surprise, we noticed that the total NPC number dramatically increased from an average of 3000 to almost 5000 per nucleus in Tpr-depleted cells (Fig. 1A,B; Supplemental Fig. S1A). This phenomenon was observed using two different siRNAs, reducing the likelihood of an off-target effect (Fig. 1C). Strikingly, in some cells, we were able to detect close to 8000 NPCs—a number typically not observed in this cell line (Fig. 1C). To our knowledge, this is the first and potentially only Nup causing this effect when depleted (Fig. 1A). Tpr knockdown using either of the two siRNAs did not arrest the cell cycle progression (Supplemental Fig. S1B, top panel), suggesting that the NPC number increase was not the result of cells spending more time in S phase, the time during which NPCs double. Importantly, we found that the density of NPCs increased in Tpr-depleted cells (Fig. 1D). Next, we stained Tpr-depleted U2OS cells with the scaffold Nups such as Nup96 and Nup133 as well as Elys and Pom121. We found all of these Nups to be increased at the NE, confirming that the additional NPCs were fully assembled and did not represent assembly intermediates (Supplemental Fig. S1D–G). The effect of Tpr depletion was specific, since the expression of full-length siRNA-resistant Tpr efficiently reversed the increase in both NPC number and density (Fig. 1E,F). Similar effects were observed in HeLa cells, human IMR-90 primary fibroblasts, and human embryonic stem cells as well as mouse myoblasts (C2C12) (Fig. 1G–J; Supplemental Fig. S1H,I). Together, these findings show that Tpr is a negative regulator of NPC number across different cell types and species, highlighting an evolutionary conserved role for this Nup.

### Nup153 phosphorylation is down-regulated in Tpr-depleted cells

**Figure 2.**
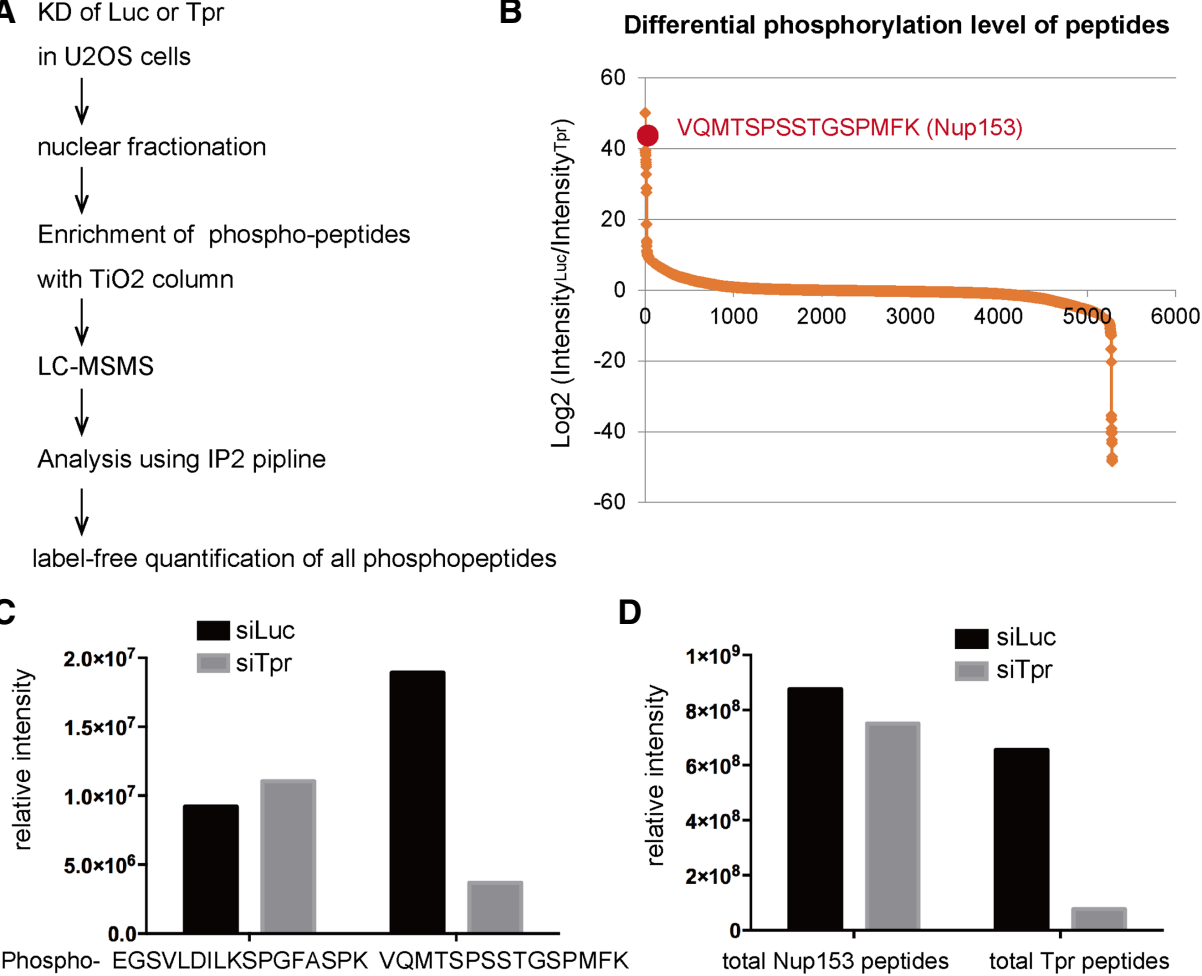
Phosphoproteomics revealed decreased phosphorylation of Nup153 in Tpr knockdown cells. (*A*) The workflow of phosphoproteomics. (*B*) Label-free quantification of all of the phosphopeptides obtained (5282 phosphopeptide species) was performed. The intensity based on the peak areas of phosphopeptides was calculated with Skyline pipeline. The intensity in the control knockdown condition was divided by the intensity of the same phosphopeptide in the Tpr knockdown condition to obtain differential phosphorylation levels between two conditions (i.e., decreased phosphorylation in the Tpr knockdown condition). Phosphopeptides were ranked according to log_2_ ratio. The *Y*-axis indicates log_2_ value. (*C*) Label-free quantification of two different phosphopeptides derived from Nup153 was performed by Skyline. The *Y*-axis indicates total signal intensity. (*D*) The amount of total peptides that represents the protein level was analyzed using Skyline pipeline. All of the peptides derived from either Nup153 or Tpr before phosphopeptide enrichment were quantified and summarized. The total abundance of Nup153 peptides did not change in both knockdown conditions, while Tpr peptides were significantly reduced in Tpr knockdown condition.

We next wanted to determine the mechanism by which Tpr depletion results in the dramatic increase in NPC numbers per nucleus. We did not observe a major change in the overall protein levels of Nups in Tpr knockdown cells (Supplemental Fig. S1C), although there were more NPCs present in the NE. This suggested that not the production but the assembly of Nups was affected. We therefore focused on post-translational modifications (particularly phosphorylation), which have been shown to play important roles in NPC biogenesis. For example, in mitosis, hyperphosphorylation of Nup98 triggers the NPC disassembly (Laurell et al. 2011; Linder et al. 2017; Martino et al. 2017), while, in interphase, two cyclin-dependent kinases (Cdks)—Cdk1 and Cdk2—are required for the NPC assembly (Maeshima et al. 2010). To obtain an unbiased view of NPC protein phosphorylation, we decided to investigate the phosphorylation status of Nups in Tpr-depleted conditions. To do this, we extracted a nuclear fraction from Tpr-depleted U2OS cells and performed mass spectrometry-based phosphoproteomics (Fig. 2A). We successfully obtained ∼5000 different phosphopeptides. We performed a label-free quantification for all of the phosphopeptides that we obtained and analyzed the differential phosphorylation levels between the control knockdown condition and the Tpr knockdown condition. Surprisingly, when we ranked the phosphopeptides according to the decrease level in Tpr knockdown condition, we noticed that phosphorylation of one peptide (VQMTSPSSTGSPMFK) from Nup153 showed one of the largest decreases among all of the phosphopeptides (Fig. 2B). Importantly, the total amount of Nup153 peptides and a different phosphopeptide (EGSVLDILKSPGFASPK) from Nup153 did not change (Fig. 2C,D), suggesting that the decreased phosphopeptide is not due to a reduction in Nup153 protein levels. This was further supported by Western blotting analysis, which showed no change in protein levels of Nup153 in Tpr knockdown cells (Supplemental Fig. S1C). Two independent phosphoproteomic experiments were performed and confirmed these findings (Supplemental Fig. S2). We conclude that Tpr is required for the phosphorylation of Nup153 at the region VQMTSPSSTGSPMFK.

### ERK phosphorylates Nup153 and negatively regulates NPC numbers

**Figure 3.**
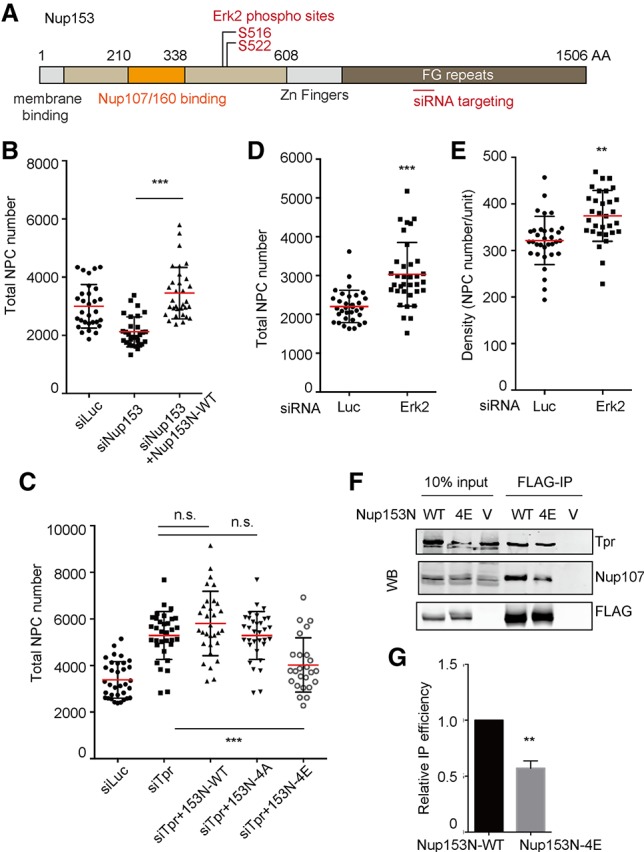
Nup153 phosphorylation by Erk negatively regulates NPC number. Mean values of NPC numbers are indicated by red bars. *t*-test was performed to obtain *P*-value. (**) *P* < 0.001; (***) *P* < 0.0001. More than 20 nuclei were used to obtain the total NPC number or the density in each condition. (*A*) A schematic diagram of human Nup153. (*B*) Nup153-N wild type was transfected with siRNA against endogenous Nup153 to confirm that the N-terminal potion of Nup153 is sufficient to assemble NPCs. siRNA against endogenous Nup153 and an N-terminal Nup153 fragment was cotransfected in U2OS cells. Sixty hours after transfection, cells were fixed and stained with Elys, and NPC numbers were counted using SR-SIM. (*C*) Nup153-N mutants were cotransfected with siRNA against Tpr. Sixty hours after transfection, cells were fixed and stained with Elys. NPC numbers and densities were obtained by counting Elys foci using SR-SIM. (*D*,*E*) The numbers of Elys foci were counted using SR-SIM in Erk2-depleted U2OS cells. (*F*) Immunoprecipitation using Flag tag of Nup153-N constructs was performed. HEK293T cells were transfected with Nup153-N constructs. Seventy-two hours after transfection, cells were harvested and used for immunoprecipitation. Western blotting was performed for the precipitated proteins. (WT) Nup153-N wild type; (5A) Nup153-N with alanine mutations; (5E) Nup153-N with glutamic acid mutations; (V) a vector control (pCDNA3.1 vector). (*G*) Quantification of immunoprecipitation–Western blotting of Nup153-N constructs and Nup107 was performed using ImageJ software.

Nup153 has been shown recently to play a key role in interphase NPC assembly by recruiting crucial core NPC component the Nup107/160 complex to the INM (Supplemental Fig. S5A). We wished to know whether the phosphorylation of the region VQMTSPSSTGSPMFK in Nup153 plays a role in determining NPC number. To test this, we generated the 1- to 608-amino-acid N-terminal construct of Nup153 (Nup153-N), whose four serines (Ser516, Ser518, Ser519, and Ser522) were mutated to either phosphorylation-deficient residues (Ala; named Nup153-N-4A) or phosphorylation-mimetic residues (Glu; named Nup153-N-4E). All of these mutants properly localized at the NE (Supplemental Fig. S5B). Nup153-N has all of the necessary domains for NPC assembly (membrane binding and Nup107/160 binding as well as the ERK phosphorylation site) (Fig. 3A). We therefore reasoned that Nup153-N should be able to rescue the NPC assembly defect observed in Nup153 knockdown cells. Notably, siRNA against endogenous Nup153 does not hit Nup153-N (Fig. 3A). Indeed, Nup153-N was able to rescue the NPC number decrease in Nup153 knockdown cells (Fig. 3B), suggesting that this part on Nup153 is sufficient for NPC assembly. We observed that Nup153 phosphorylation is down-regulated in the Tpr knockdown condition, which causes NPC number increase (Fig. 2B–D; Supplemental Fig. S2). We hypothesized that if Nup153 phosphorylation negatively regulates NPC assembly, then phosphorylation-mimetic Nup153-N-4E should be able to reverse NPC number increase in Tpr knockdown cells by bypassing the lack of its phosphorylation. To test this, we transfected siRNA against Tpr together with Nup153-N mutant constructs. Strikingly, Nup153-N-4E was able to rescue the NPC number increase caused by Tpr knockdown, while the phosphorylation-deficient Nup153-N-4A was not (Fig. 3C). We concluded that phosphorylation of Nup153 is required to negatively regulate its NPC assembly ability.

We next wondered what the responsible kinase for Nup153 phosphorylation is. Interestingly, the phosphopeptide (VQMTS*PSSTGS*PMFK, where the asterisk indicates a known ERK phosphorylation site) was shown previously to be phosphorylated by ERK (Kosako et al. 2009). ERK is a major MAPK that regulates many important biological processes such as proliferation, differentiation, or survival, responding to various kinds of stimulations (Roberts and Der 2007). We therefore decided to apply RNAi-mediated knockdown of the major ERK: ERK2. If ERK2 is the responsible kinase for Nup153 phosphorylation and therefore the negative regulation of NPC assembly, then ERK2 depletion should result in an increase in NPC numbers. We depleted ERK2 in U2OS cells and monitored an increase in NPC number and density (Supplemental Fig. S3A; Fig. 3D,E). As ERK2 knockdown did not arrest the cell cycle, the increase in NPC number was not due to the extended interphase period, which allows cells to insert more NPCs (Supplemental Fig. S3B). The NPC density also increased (Fig. 3E), providing evidence that the increased NPC number is not due to nuclear growth. These results suggest that ERK2, like Tpr, negatively regulates NPC number.

It has been shown that Nup153 binds and recruits the essential NPC core component the Nup107/160 complex to the INM (Vollmer et al. 2015). Thus, we wished to test whether the phosphorylation of Nup153 by ERK directly regulates its interaction with the Nup107/160 complex. To test this, we expressed the C-terminally Flag-tagged Nup153-N mutants in HEK293T cells and investigated the interaction with Nup107 by Flag immunoprecipitation. We found that wild-type Nup153-N precipitated endogenous Nup107 as well as endogenous Tpr (Fig. 3F). However, the phosphorylation-mimetic Nup153N-E mutant precipitated significantly less Nup107 compared with wild type, while the amount of precipitated Tpr did not change between the two constructs (see Fig. 3F,G for quantification). This suggests that phosphorylation of Nup153 reduces the interaction specifically with Nup107. Furthermore, as the amount of precipitated Tpr did not change, we concluded that the Nup153 binding to the fully assembled NPCs was not likely affected by phosphorylation. Our observations strongly support the idea that ERK phosphorylates Nup153 to negatively regulate its ability to recruit Nup107 to NPC assembly sites.

### Erk phosphorylated Tpr serves as a scaffold for ERK to regulate NPC numbers

**Figure 4.**
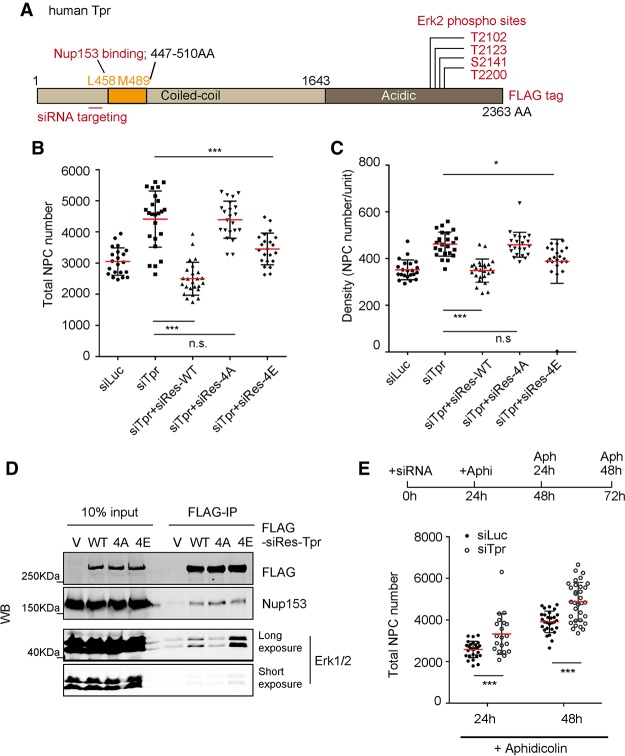
Tpr phosphorylation by Erk is required for NPC number regulation. Mean values of NPC numbers are indicated by red bars. *t*-test was performed to obtain *P*-value. (*) *P* < 0.01; (***) *P* < 0.0001. More than 20 nuclei were used to obtain the total NPC number or the density in each condition. (*A*) A schematic diagram of human Tpr. (*B*,*C*) siRNA against endogenous Tpr was transfected to U2OS cells that stably expressed siRNA-resistant Tpr mutants. Seventy-two hours after transfection, total NPC number and the density were obtained by counting Nup153 foci using SR-SIM. (*D*) Immunoprecipitation using Flag tag of siRes-Tpr constructs was performed. HEK293T cells were transfected with siRes-Tpr constructs. Seventy-two hours after transfection, cells were harvested and used for immunoprecipitation. Western blotting was performed for the precipitated proteins. (WT) siRNA-resistant Tpr wild type; (4A) siRNA-resistant Tpr with alanine mutations; (4E) siRNA-resistant Tpr with glutamic acid mutations; (V) a vector control (pQCXIB-CMV-TO-Dest). (*E*) Cells were treated with 10 µM aphidicolin after 24 h of knockdown. Cells were cultured in the presence of aphidicolin for either 24 or 48 h. Cells were fixed and stained with Nup153 and then analyzed by SR-SIM. More than 20 nuclei were analyzed to obtain the total NPC number in each condition.

Our results suggest that Tpr regulates ERK-mediated phosphorylation of Nup153 to negatively regulate NPC assembly. How, then, does Tpr regulate Nup153 phosphorylation by ERK? Interestingly, we found one of the serines in the C terminus of Tpr to be specifically phosphorylated by ERK (TDGFAEAIHS*PQVAGVPR, where the asterisk indicates the ERK phosphorylation site) (Fig. 4A, S2141). It was reported previously that ERK phosphorylates three threonine residues and one serine residue in the C terminus of Tpr (Fig. 4A). Surprisingly, these phosphorylated residues of Tpr by ERK were shown to stabilize the Tpr–ERK interaction (Vomastek et al. 2008), which is unusual, as phosphorylation typically destabilizes the interaction between ERK and the substrates. Given that Nup153 phosphorylation decreases in Tpr knockdown condition, we speculated that ERK, which stably binds to the NPC through phosphorylated Tpr, phosphorylates Nup153. Supporting this notion, our experiment showed that knockdown of Tpr caused the decrease of Nup153 phosphorylation (Fig. 2B,C; Supplemental Fig. S2), presumably because ERK lost its ability to bind NPCs and thus no longer was able to phosphorylate Nup153. We therefore hypothesized that a Tpr–ERK complex regulates the NPC number through Nup153 phosphorylation. To test this idea, we first wished to determine whether phosphorylation of Tpr stabilizes ERK binding as reported previously. To do this, we decided to mutate all four residues of ERK targeting sites in Tpr to either phosphorylation-deficient (Ala) or phosphorylation-mimetic (Glu) residues (referred to as Tpr-4A and Tpr-4E, respectively). Each mutant contains a Flag tag at the C-terminal. We observed that these mutants properly localize at the NE, indicating that incorporation into NPCs is not compromised (Supplemental Fig. S4A). We transiently expressed these mutants in HEK293T cells and performed immunoprecipitation using Flag tag. We found that the Tpr-4E mutant efficiently precipitates ERK1/2, while the Tpr-4A mutant hardly precipitates ERK1/2 (Fig. 4D). Considering that the previous report showed that phosphorylation-deficient mutations of the four residues reduce Tpr–ERK interaction (Vomastek et al. 2008), by using the Tpr-4E mutant, our result further strengthens their finding that Tpr phosphorylation indeed stabilizes Tpr–ERK interaction.

To further confirm the link between the Tpr–ERK complex and the NPC number, we decided to investigate whether Tpr phosphorylation by ERK is required for NPC number regulation. We stably expressed the phosphorylation mutants of Tpr as described above in U2OS cells. Each mutant also carried siRNA-resistant mutations to allow us to analyze whether the mutants can rescue the Tpr knockdown phenotype when endogenous Tpr is depleted by siRNA. As observed previously, siRNA-resistant wild-type Tpr was able to rescue the NPC number and density increase caused by the depletion of endogenous Tpr (Fig. 4B,C). Strikingly, we found that a phospho-mimetic mutant (Tpr-4E), but not a phospho-deficient mutant (Tpr-4E), was able to rescue both the NPC number increase and the density increase (Fig. 4B,C). This observation strongly supports the idea that the Tpr–ERK complex negatively regulates NPC numbers. A previous study showed that Tpr–ERK interaction is required for ERK translocation into the nucleus (Vomastek et al. 2008). However, we did not observe a major change in the nucleo–cytoplasmic distribution of ERK or phosphorylated ERK in Tpr knockdown cells (Supplemental Fig. S3E). This suggests that nuclear transport of ERK is not responsible for NPC number regulation. Given these results, we concluded that the Tpr–ERK complex negatively regulates NPC numbers by phosphorylating Nup153 in order to negatively regulate the Nup153 function of recruiting NPC core components to the NPC assembly sites.

### Tpr is a negative regulator of interphase NPC assembly

In cycling cells, NPC assembly occurs at two different stages. One is a rapid assembly at the exit of mitosis (post-mitosis), and the other occurs during interphase, when cells double the NPC numbers between G1 and G2. Previous reports showed that Nup153 is not required for post-mitotic NPC assembly (Walther et al. 2001; Vollmer et al. 2015). This implies that the Tpr–ERK complex regulates interphase NPC assembly rather than post-mitotic NPC assembly. To directly test this idea, we decided to investigate whether the Tpr–ERK complex can regulate NPC assembly without going through mitosis. We arrested control and Tpr-depleted U2OS cells in G1/S phase using aphidicolin for 2 d (Fig. 4E, top panel; Supplemental Fig. S1A, bottom panel). In this prolonged G1/S arrest, we observed an increase of NPC number in control cells as cells kept assembling NPCs (Fig. 4E). Importantly, depletion of Tpr under these conditions resulted in a dramatic increase in NPC numbers (Fig. 4E). This suggests that Tpr depletion causes excessive NPC assembly in interphase in the absence of post-mitotic NPC assembly and that Tpr negatively regulates interphase NPC assembly.

### The Tpr–ERK complex regulates NPC numbers at the NE

**Figure 5.**
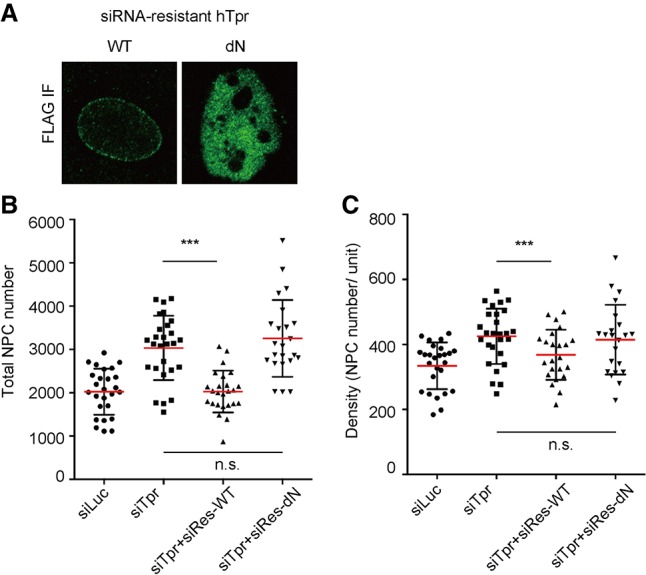
Tpr regulates NPC number at the NE. Mean values of NPC numbers are indicated by red bars. *t*-test was performed to obtain *P*-value. (***) *P* < 0.0001. More than 20 nuclei were used to obtain the total NPC number or the density in each condition. (*A*) The localization of siRNA-resistant Tpr constructs was investigated by immunofluorescence using Flag tag. The dN mutant, which carries two point mutations in the Nup153-binding site (L458D and M489D), cannot localize at the NE due to the lack of Nup153-binding ability. (*B*,*C*) siRNA against endogenous Tpr was transfected to U2OS cells, which stably expressed the siRNA-resistant Tpr mutant. Seventy-two hours after transfection, total NPC number and the density were obtained by counting Nup153 foci using SR-SIM.

We showed that Tpr and Erk form a complex to phosphorylate Nup153. Nup153 is one of the dynamic Nups, which can shuttle between the nucleoplasm and the cytoplasm. In addition, Tpr was shown to be present in filamentous structures that extend into the nucleoplasm (Cordes et al. 1997; Fontoura et al. 2001). Considering these findings, we were curious to directly test whether Erk binds to Tpr at NPCs. We performed the proximity ligation assay between Erk and exogenously expressed Tpr. Interestingly, although we observed nucleoplasmic interactions, the interaction between these two proteins occurs at the NE, similar to the pattern observed for Tpr–Nup153 interactions (Supplemental Fig. S4D). This strongly supports the idea that Erk can bind to Tpr that has been incorporated into the NPC basket. To further confirm that NPC-bound Erk regulates NPC assembly, we wanted to know whether NPC association of the Tpr–ERK complex is required to regulate NPC numbers. To answer to this question, we expressed an siRNA-resistant version of Tpr that carries two amino acid mutations in the Nup153-binding domain (L458D and M489D) that disrupt its ability to bind Nup153 (Cordes et al. 1998; Hase et al. 2001). Since Nup153 anchors Tpr to the NPC, we found that this construct localized in the nucleoplasm (Fig. 5A), as reported previously (Hase and Cordes 2003). Interestingly, we found that this mutant was not able to rescue the NPC number increase, while the wild type was able to do so (Fig. 5B,C). This strongly implies that the presence of Tpr at the NPCs is required for negative NPC number regulation and that ERK regulates NPC numbers by binding to the NPCs at the NE.

## Discussion

### The Tpr–ERK complex as a negative feedback regulator of NPC assembly

Tpr is a 267-kDa Nup that forms a homodimer that is believed to represent the spokes of the eightfold basket filaments of NPCs (Krull et al. 2004). Like other Nups, Tpr can bind to chromatin and regulate chromatin organization, such as heterochromatin-excluded zones (Krull et al. 2010; Vaquerizas et al. 2010; Ibarra and Hetzer 2015). Here we provide evidence of a new function of Tpr as a negative regulator of NPC assembly. The protein levels and phosphorylation levels of Tpr determine how many NPCs are assembled in a given cell. In its absence, NPC numbers and NPC density in the NE increase. This mechanism is mediated through ERK phosphorylation of Nup153, which interferes with its recruitment of the Nup107/160 complex. Our results suggest that Tpr is part of a negative feedback loop in cycling cells to control the number of NPCs. We propose a model in which the increase in NPC numbers results in an increase of Tpr–ERK complexes at the NE (Supplemental Fig. S5B). This results in elevated levels of phosphorylation of Nup153 in proximity to already assembled NPCs, which in turn results in a repression of NPC assembly at the NE (Supplemental Fig. S5B). We suggest that the ERK pathway initially stimulates NPC assembly, as evidenced by the previous work showing that mechanical NE stretching increased the protein level of Nups as well as NPC number through the ERK pathway (Richard et al. 2007). On the other hand, the phosphorylation of Tpr by ERK takes place as the ERK pathway is turned on. When there are not enough NPCs assembled on the nuclear surface (for example, in early interphase), there would not be enough Tpr–ERK complexes to suppress NPC assembly events happening across the nuclear surface (Supplemental Fig. 5B, panel I). As NPC assembly progresses, the local concentration of Tpr–ERK complexes increases to levels that are sufficient to suppress NPC assembly (Supplemental Fig. S5B, panel II). It remains to be determined whether Tpr plays a role in NPC number regulation during early developing embryos. Embryos at blastula stage undergo very rapid cell divisions and have to assemble new NPCs in a short period of time. It also will be interesting to test whether the number of NPCs is regulated in the *Drosophila* syncytial blastoderm, where preassembled NPCs in the ER membrane (annulate lamellae) are inserted into the NE (Hampoelz et al. 2016).

### The phosphorylation of Nups by the Tpr–ERK complex

Our data clearly show that ERK has to associate with the NPC in order to regulate Nup153 phosphorylation (Fig. 5). Due to technical limitations, we were not able to determine exactly when Nup153 phosphorylation by the Tpr–ERK complex occurs. Since Nup153 is a dynamic Nup that shuttles between the cytoplasm and the nucleoplasm, it is possible that newly synthesized Nup153 protein might be phosphorylated by the Tpr–ERK complex when it passes through NPCs during nuclear import. Importantly, phosphorylated Nup153 is no longer able to recruit the Nup107/160 complex to the NE, thereby preventing new NPCs from being assembled into the NE. In addition to Nup153, one of the early players of interphase NPC assembly is a membrane Nup: Pom121 (Doucet et al. 2010). Unfortunately, our phosphoproteomics was not able to detect Pom121. New approaches will have to be developed to investigate whether Pom121 and other Nups, which have been shown to be phosphorylated by ERK (Kosako et al. 2009), can be phosphorylated by the Tpr–ERK complex. Notably, the NPC basket is a multifunctional structure that is engaged in nuclear transport as well as transcription/chromatin organization. Anchoring ERK at the NPC basket might be an important spatial aspect of nuclear organization that would allow ERK to directly regulate these essential processes, responding to extracellular stimuli. The important observation made in yeast is that yeast Tpr Mlp1/2 anchors Mad1–Mad2 kinases at the NPCs in interphase in order to inhibit abnormal anaphase progression in coming mitosis (Rodriguez-Bravo et al. 2014). The NPC basket structure might serve as a central scaffold for various important signaling pathways.

### The biological importance of NPC number regulation

Our study provides direct evidence that the NPC assembly process is under negative control. At this point, we can only speculate why cells regulate the total number of NPCs per nucleus and what the consequence of increased NPC numbers might be. It has been shown that the cells with higher metabolic activity, such as stimulated thyroid follicular cells or aggressive tumors, have more NPCs per nucleus (Maul et al. 1971; Many et al. 1981). It is possible that higher NPC numbers reflect increased nucleo–cytoplasmic transport capacity. Consistent with this idea is the finding that inhibition of nuclear export factors by small molecules shows dramatic effects in cancer treatment (Kim et al. 2016). Furthermore, aggressive cancer cells often acquire multidrug resistance. It was shown that the multidrug resistance is related to the higher numbers of NPCs, as the cells are more capable of exporting the drugs from the nuclei (Lewin et al. 2007). In addition to nuclear transport, NPCs have also been shown to regulate transcription. Most notably, NPC components have been shown to bind superenhancers (SEs)—regulatory structures that drive the expression of key genes that specify cell identity (Ibarra et al. 2016). It is possible that changes in NPC numbers might result in transcriptional changes of SE-associated genes. Furthermore, mutations in the ERK pathway are frequently associated with tumorigenesis. It will be interesting to study whether and how Tpr-mediated NPC assembly affects cell transformation. Finally, NPCs are shown to be very long-lived protein structures whose components can last for years in terminally differentiated cells such as neurons (Savas et al. 2012; Toyama et al. 2013). It is possible that in post-mitotic cells, NPCs might play a role in maintaining the three-dimensional chromatin structures and epigenetics of cell identity genes and thus the maintenance of the functions of terminally differentiated cells.

## Materials and methods

### Cell lines and transfections

U2OS cells and HeLa cells were cultured in DMEM (Gibco) and 10% FBS. C2C12 cells were cultured in DMEM and 20% FBS. IMR90 cells were cultured in DMEM and 20% FBS supplemented with nonessential amino acids (Gibco) and low-oxygen conditions (3%). siRNA-mediated gene silencing was performed using the oligos (Invitrogen) control (Luc, 5′-UAUGCAGUUGCUCUCCAGC-3′), human Tpr (5′-UUUAACUGAAGUUCACCCU-3′), mouse Tpr (5′-AUACCGCAAACUCUCAACCTT-5′), and human Erk2 (5′-GGUGUGCUCUGCUUAUGAU-3′) (von Thun et al. 2012) and delivered using siLentFect (Bio-Rad). siRNAs against human Nup153, Nup107, Elys, Nup98, and Pom121 were designed according to previous studies (Doucet et al. 2010; Ibarra et al. 2016). DNA transfection was carried out using Lipofectamine 2000 (Invitrogen). Cotransfection of DNA and siRNA was carried out using Lipofectamine 2000 with 50 nM siRNA and 1 ng/µL plasmid in final concentration.

### Plasmids

pEGFP-N1-Tpr (from Dr. Larry Gerace [Addgene plasmid, 35024]) was purchased from Addgene. siRNA-resistant full-length Tpr mutants were generated using the combination of the Gibson assembly method and a site-directed mutagenesis method. N-terminal and C-terminal fragments of hTpr (N-terminal: 1–2400 base pairs [bp]; C-terminal: 4878–7050 bp) were amplified by KOD polymerase using pEGFP-N1-hTpr plasmid. N-terminal (Tpr-N) and C-terminal (Tpr-C) fragments were cloned into pUC19 vector using KpnI/EcoRI and HindIII/SalI. siRNA-resistant mutations or phosphorylation mutations were introduced into pUC19-Tpr-N or pUC19-Tpr-C by a site-directed mutagenesis method. Next, mutant Tpr-N, mutant Tpr-C, and the middle region of Tpr (2401–4877 bp) were amplified by PCR to generate fragments ready for the seamless cloning method Gibson assembly. At the same time, PCR was performed to generate a fragment corresponding to the pQCXIB-TO-CMV retrovirus vector. The sequences of the primer sets were designed using NEBuilder assembly tool (http://nebuilder.neb.com/ NEB). Four fragments were assembled using Gibson assembly master mix (New England Biolabs) following the directions. The resultant products (pQCXIB-full-length hTpr) were amplified in Stbl3 bacteria at 30°C. The N-terminal portion of human Nup153 was PCR-amplified with Flag tag sequences at its C-terminal end and cloned into the pUC19 vector using HindIII and BamHI. Phosphorylation mutations were introduced by a site-directed mutagenesis method. Nup153-N fragments were cut out using the same restriction enzyme sites and ligated into the pCDNA3.1 vector.

### Immunofluorescence and antibodies

For immunofluorescence assays, cells were fixed with 2% paraformaldehyde (PFA) for 10 min. After fixation, cells were washed three times with PBS(−) and permeabilized and blocked for 20 min in immunofluorescence buffer (10 mg/mL BSA, 0.1% Triton X-100, 0.02% sodium dodecyl sulfate [SDS], 1× PBS). Next, cells were incubated for 1 h in immunofluorescence buffer containing antibodies. After three washes with PBS(−), cells were incubated with Alexa fluor-conjugated secondary antibodies. Cells were mounted using VectaShield (Vector Laboratories) or ProLong Gold anti-fade mountant (Thermofisher). The antibodies used were as follows: Tpr (1:500 dilution; Abcam, ab84516), Pom121 (1:500; Genetex, GTX102128), Nup153 (SA1 mouse ascites from Dr. B. Burke), Elys (1:1000; produced in the Mattaj laboratory, EMBL), Nup133 (1:750; produced in the Hetzer laboratory), Nup96 (1:500; Novus Biologicals, NB100-93325), Flag (M2) (1:1000; Sigma, F3165), hErk2 (for Western blotting, 1:1000; Abcam, E460), phospho-Erk1/2 (1:1000; Cell Signaling 4376), and Erk1/2 (1:1000, Cell Signaling, [137F5] 4695). EdU labeling and the visualization were performed according to manufacturer's instructions (Click-iT EdU imaging kit, Invitrogen, C10338).

### Imaging

Confocal microcopy was performed on a Zeiss LSM710. Images were processed using ImageJ. SR-SIM was performed on Zeiss Elyra PS.1 using a 63× (oil) objective lens. After a reconstruction by Zen software, NPC numbers were counted using a Spot function of Imaris software by excluding foci <0.09 nm.

### Immunoprecipitation

HEK293T cells were transfected with pCDNA3.1-Nup153-N constructs or pQCXIB-Tpr constructs. Seventy-two hours after transfection, cells were harvested with trypsin. Cells were washed with PBS once and then lysed into 1 mL of buffer-1 (150 mM NaCl, 10 mM Tris-Cl at pH 7.5, 0.1% Triton X-100, 1 mM EDTA, 0.25% NaDoC, Complete EDTA-free [Themofisher], 1 mM DTT). After passing through a 25-gauge needle 10 times, cell lysates were centrifuged at 20,000 relative centrifugal force (rcf) for 10 min. The supernatants were added to 50 µL of Flag-M2 agarose beads (Sigma). After rotation for 2 h at 4°C, lysates were aspirated and washed five times with buffer-1. Proteins were eluted from beads by adding 100 µL of 2× SDS sample buffer and incubating for 5 min at 95°C. Twenty microliters of the eluted proteins were run on 6% or 8% SDS-PAGE.

### Cell cycle arrest

U2OS cells or HeLa cells were treated with 10 µM aphidicolin. For the prolonged G1/S arrest, cells were treated with 10 µM aphidicolin after 24 h of knockdown. Cells were further cultured in the presence of aphidicolin for either 24 or 48 h.

### Proximity ligation assay

Proximity ligation assay was performed using Duolink Proxomity ligation assay kit (Sigma). To analyze the interaction between endogenous Nup153 and endogenous Tpr, we used antibodies against those proteins described above. siRNA-resistant hTpr was transiently transfected to analyze the interaction between Tpr and Erk1/2. An antibody against Flag tag (Sigma, M2) and an antibody against Erk1/2 (Abcam, ab184699) were used.

### Mass spectrometry

Samples were precipitated by methanol/chloroform. Dried pellets were dissolved in 8 M urea and 100 mM TEAB (pH 8.5). Proteins were reduced with 5 mM tris(2-carboxyethyl)phosphine hydrochloride (TCEP) (Sigma-Aldrich) and alkylated with 10 mM chloroacetamide (Sigma-Aldrich). Proteins were digested overnight at 37°C in 2 M urea and 100 mM TEAB (pH 8.5) with trypsin (Promega). Digestion was quenched with formic acid at 5% final concentration. Samples were phosphoenriched with Pierce TiO_2_ phosphopeptide enrichment kit (Thermo, 88301) per kit instructions.

The digested samples were analyzed on a Fusion Orbitrap tribrid mass spectrometer (Thermo). The digest was injected directly onto a 30-cm, 75-µm internal diameter column packed with BEH 1.7-µm C18 resin (Waters). Samples were separated at a flow rate of 300 nL/min on a nLC 1000 (Thermo). Buffers A and B were 0.1% formic acid in water and acetonitrile, respectively. A gradient of 1%–30% B over 90 min, an increase to 40% B over 30 min, and an increase to 90% B over another 10 min, held at 90% B for a final 10 min of washing, were used for a 140-min total run time. A 240-min gradient was also used, a gradient of 1%–25% B over 160 min, an increase to 35% B over 60 min, and an increase to 90% B over another 10 min, held at 90% B for a final 10 min of washing, were used for a 240-min total run time. The column was re-equilibrated with 20 µL of buffer A prior to the injection of the sample. Peptides were eluted directly from the tip of the column and nanosprayed directly into the mass spectrometer by application of 2.5 kV at the back of the column. The Orbitrap Fusion was operated in a data-dependent mode. Full MS1 scans were collected in the Orbitrap at 120,000 resolution with a mass range of 400–1600 *m/z* and an AGC target of 5e5. The cycle time was set to 3 sec, and, within the 3 sec period, the most abundant ions per scan were selected for CID tandem mass spectrometry in the ion trap with an AGC target of 1e4 and minimum intensity of 5000. Maximum fill times were set to 50 and 100 msec for mass spectrometry and tandem mass spectrometry scans, respectively. Quadrupole isolation at 1.6 *m/z* was used, monoisotopic precursor selection was enabled, and dynamic exclusion was used with an exclusion duration of 5 sec.

Protein and peptide identification were done with Integrated Proteomics Pipeline (Integrated Proteomics Applications). Tandem mass spectra were extracted from raw files using RawConverter (Schilling et al. 2012; He et al. 2015) and searched with ProLuCID (Xu et al. 2015) against the human UniProt database. The search space included all fully tryptic and half-tryptic peptide candidates. Carbamidomethylation on cysteine was considered a static modification. Phosphorylation was considered a differential modification on STY with a maximum of three per peptide. Data were searched with a 50-ppm precursor ion tolerance and a 600-ppm fragment ion tolerance and filtered to 10 ppm at the precursor after the search. Identified proteins were filtered using DTASelect (Tabb et al. 2002) and a target–decoy database search strategy to control the false discovery rate to 1% at the protein level. Peak area analysis was performed with Skyline (Schilling et al. 2012).

## Supplementary Material

Supplemental Material
