# Supplementary material for: Tpr regulates the total number of nuclear pore complexes per cell nucleus
Source: Genes Dev. 2018 Oct 1;32(19-20):1321–31. doi: 10.1101/gad.315523.118 (PMC6169833; doi:10.1101/gad.315523.118)
Supplement: Supplemental Material [file supp_32_19-20_1321__index.html]

Tpr regulates the total number of nuclear pore complexes per cell nucleus — Supplemental Material 

# Tpr regulates the total number of nuclear pore complexes per cell nucleus

## Supplemental Material

- Supplemental\_Data.pdf
