# Supplementary material for: Tpr regulates the total number of nuclear pore complexes per cell nucleus
Source: Genes Dev. 2018 Oct 1;32(19-20):1321–31. doi: 10.1101/gad.315523.118 (PMC6169833; doi:10.1101/gad.315523.118)

Figure S1. Tpr is a negative regulator of NPC numbers.

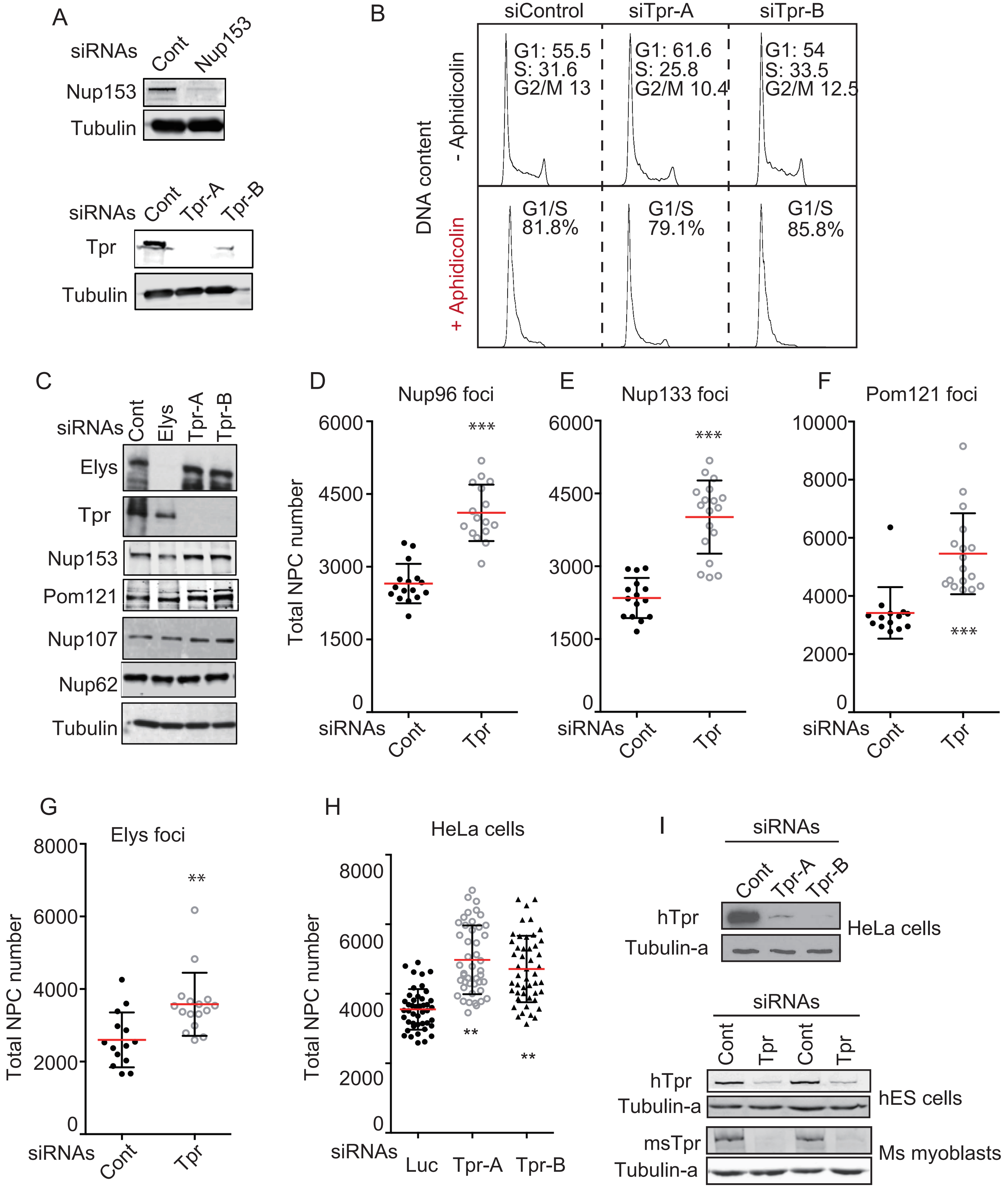

Supplementary Figure 1. Tpr is the negative regulator of NPC numbers.

Mean values of NPC numbers were indicated by red bars. t-test was performed to obtain p-value. * indicates $p<0.01$, ** indicates $p<0.001$, *** indicates $p<0.0001$. (A) knockdown of Nup153 or Tpr in U2OS cells was validated by western blotting. In the bottom panel, two different siRNAs against Tpr were used. (B) Cell cycle analysis was performed by FACS. In the top panel, U2OS cells were transfected with siRNAs and fixed with ethanol. After RNase treatment, cells were stained with PI (propidium iodide) and DNA content was analyzed by LSR II (BD). Tpr depletion did not alter the cell cycle. In the bottom panel, cells were transfected with siRNAs in the presence of Aphidicolin. (C) WB of various Nups and tubulin in Tpr or Elys depleted condition. (D)-(H) knockdown of Tpr was performed in U2OS cells. Cells were stained with various Nups and NPC numbers were obtained by counting foci of different Nups. (I) Tpr knockdown was validated in HeLa, C2C12 and human ES (H1) cells.

Figure S2. Label-free quantification of phospho-peptides from Nup153 in two experiments.

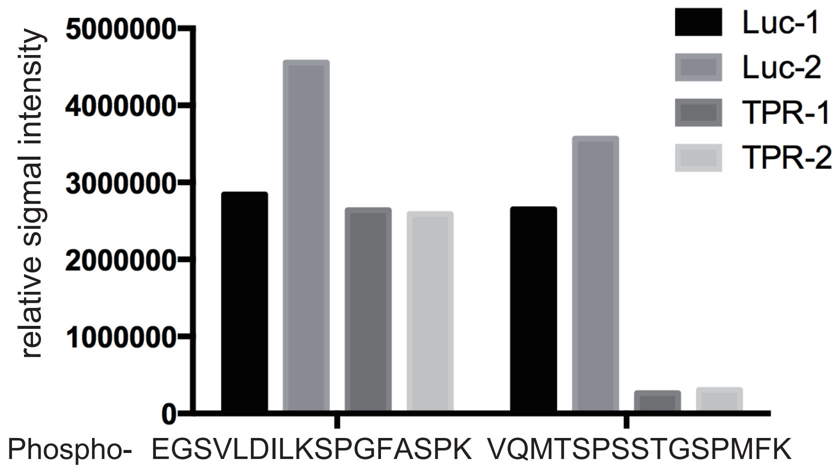

Supplementary Figure 2. Label-free quantification of phosphopeptides from Nup153 in two experiments. Phosphoproteomics in knockdown cells were performed twice. The label-free quantification of two phosphopeptides derived from Nup153 was carried out. VQMTSPSSTGSPMFK phosphopeptide showed significant decrease in Tpr knockdown condition in both experiments, while the other phosphopeptide did not change.

Figure S3. Depletion of Tpr or Erk2 in U2OS cells.

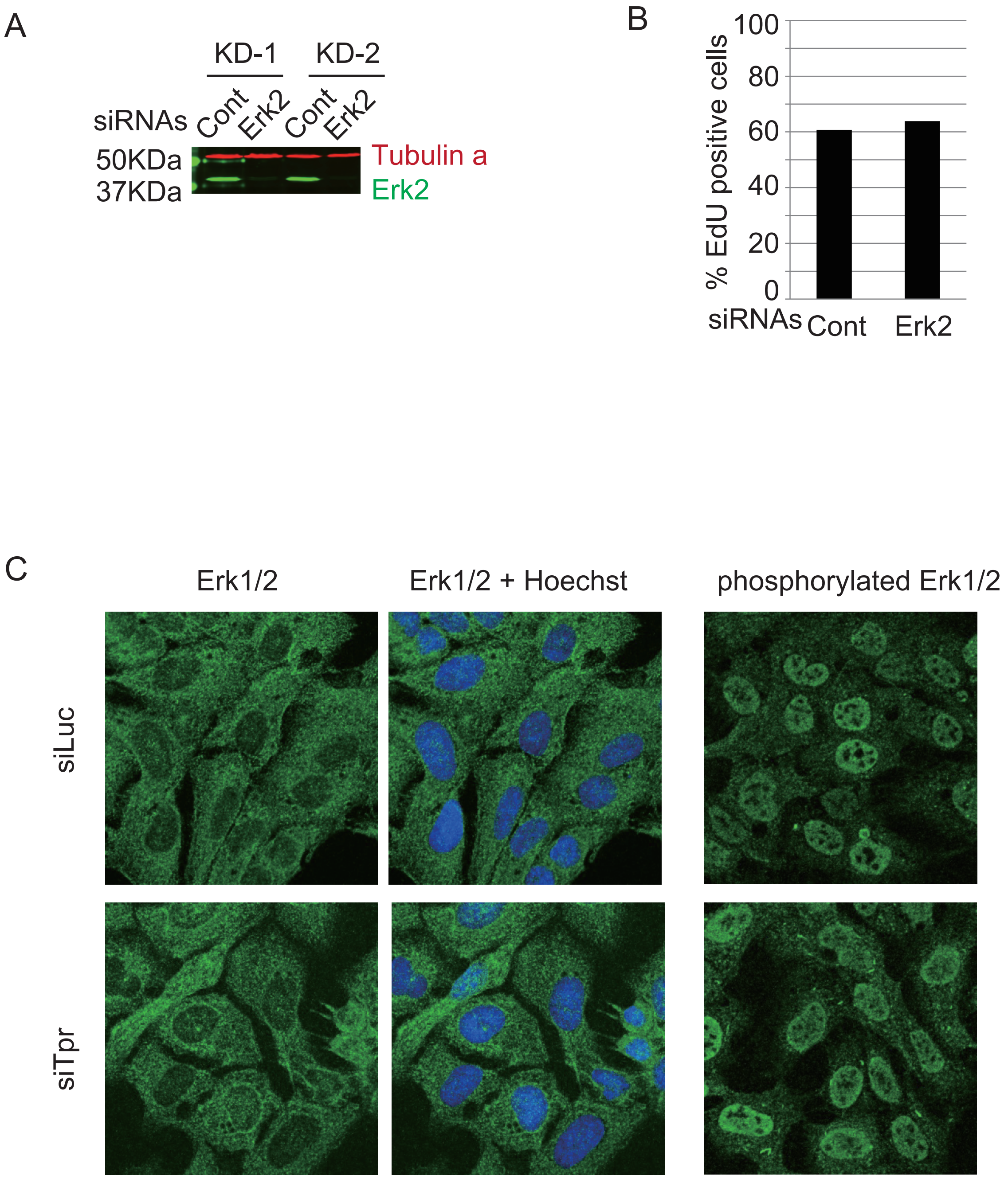

Supplementary Figure 3. Knockdown of Tpr does not change the cell cycle nor nuclear-cytoplasmic localization of ErkDepletion of Tpr or Erk2 in U2OS cells.

(A) Knockdown of Erk2 in U2OS cells was validated by western blotting. Two different knockdowns are investigated. (B) U2OS cells were labeled with 10 microM EdU for 4h and fixed after 72h of knockdown. Click reaction followed by imaging was performed according to the manufactures instructions (Invitrogen). EdU positive and negative cells were counted (N>300). (C)(B) The localization of either Erk1/2 or phosphorylated Erk1/2 was analyzed. After 72h of knockdown, U2OS cells were fixed and stained with antibodies which detect Erk1/2 or phosphorylated Erk1/2. Tpr knockdown did not change the localization of either population of Erk.

Figure S4. The localization and the interaction of recombinant mutant Nups.

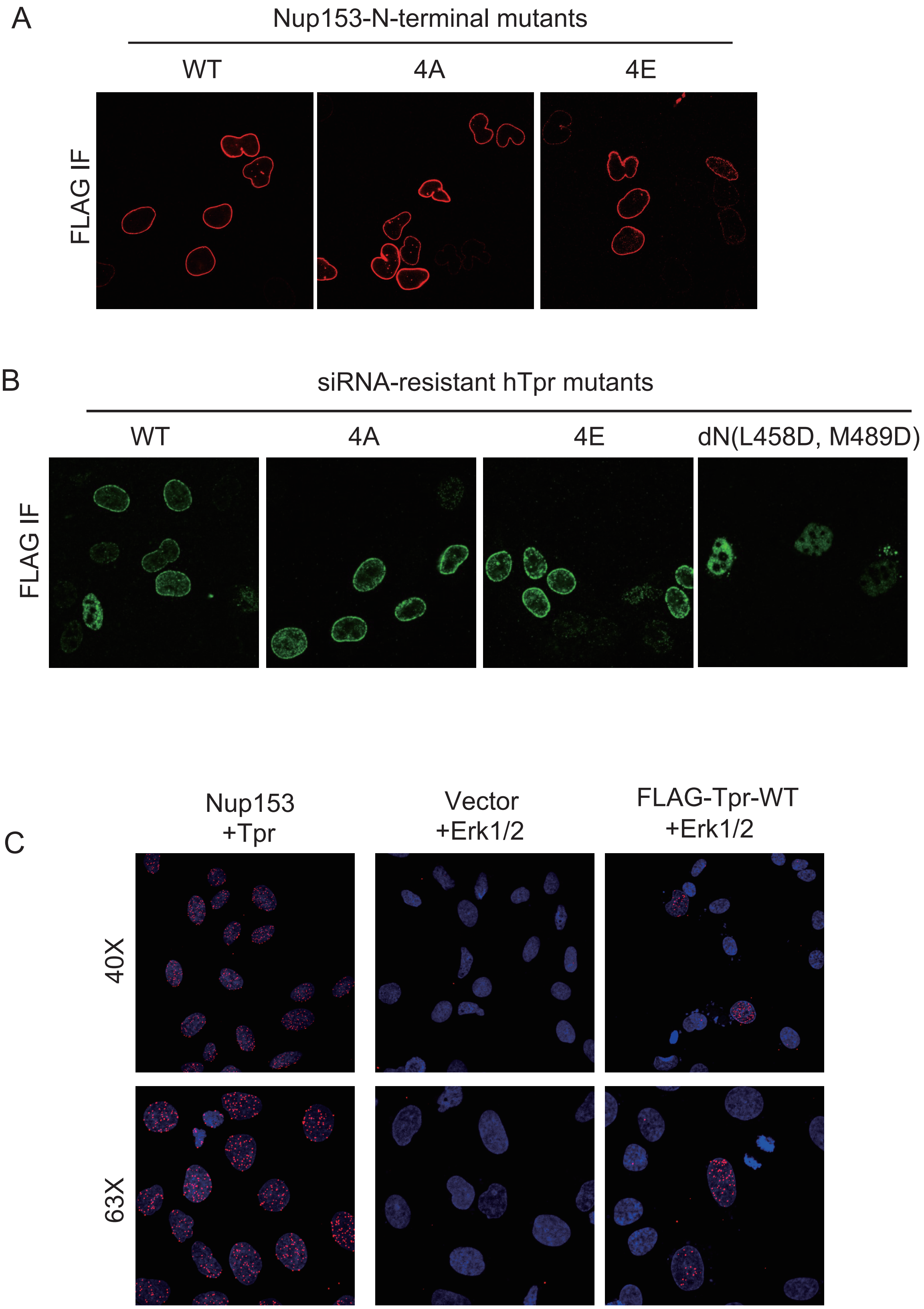

Supplementary Figure 4. The localization of recombinant mutant Nups.

(A) Nup153-N mutant constructs were transfected to U2OS cells. Cells were stained with anti-FLAG antibody. (B) siRNA-resistant Tpr constructs were transfected to U2OS cells. Cells were stained by anti-FLAG antibody. WT: full length wild-type Tpr, 4A: 4 phosphorylation sites were mutated to alanine, 4E: 4 phosphorylation sites were mutated to glutamic acid. dN: two residues in Nup153 binding site were mutated. (C) Proximity ligation assay showed that Tpr-Erk interaction occurs at the NE. The red foci indicate the interactions between proteins of interest. Upper panels: cells were observed using a 40X objective. Lower panels: Celle were observed using a 63X objective. The left column: The interaction between endogenous Nup153 and endogenous Tpr was investigated. The right column: The interaction between Flag-hTpr-WT which was transiently expressed and the endogenous Erk1/2 was investigated. The middle column: The antibody against Erk1/2 alone did not give signals.

Figure S5. Erk-Tpr complex is a part of a negative feedback of NPC assembly.

A Schematic model of interphase NPC assembly

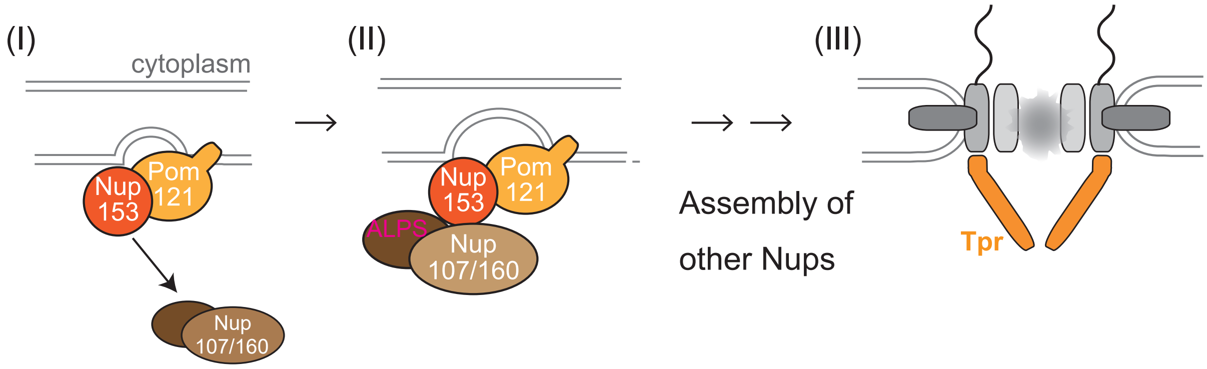

B Erk-Tpr complex is a part of a negative feedback of NPC assembly.

(I) early interphase

(II) late interphase

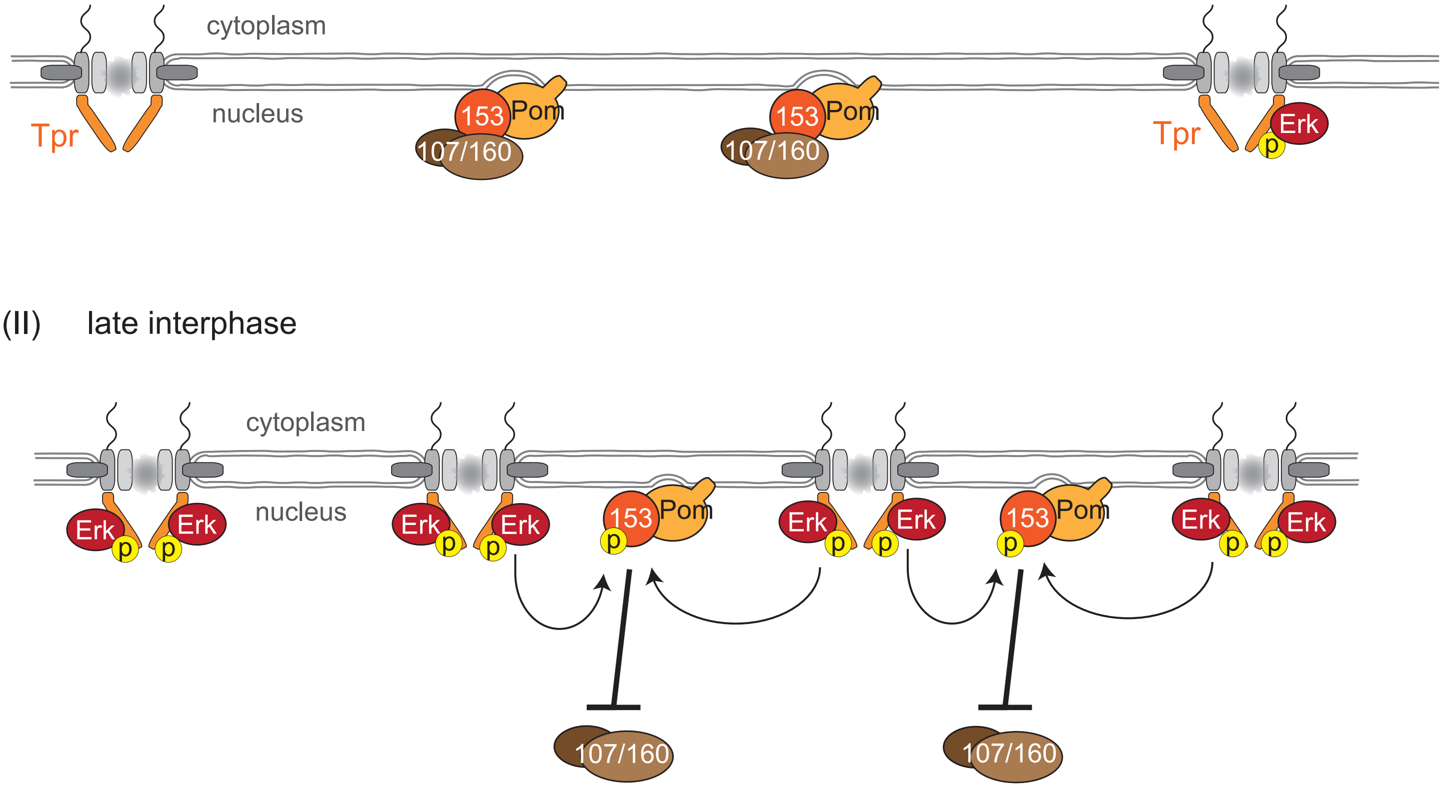

Supplement: Supplemental Material [file supp_gad.315523.118_Supplemental_Data.pdf]
